# Music evokes vicarious emotions in listeners

**DOI:** 10.3389/fpsyg.2014.00431

**Published:** 2014-05-30

**Authors:** Ai Kawakami, Kiyoshi Furukawa, Kazuo Okanoya

**Affiliations:** ^1^JST, ERATO, OKANOYA Emotional Information ProjectTokyo, Japan; ^2^Emotional Information Joint Research Laboratory, RIKEN BSISaitama, Japan; ^3^Department of Fine Arts, Tokyo University of the ArtsTokyo, Japan; ^4^Graduate School of Arts and Sciences, The University of TokyoTokyo, Japan

**Keywords:** sad music, vicarious emotion, perceived/felt emotion, ambivalent emotion, emotional quality

## Abstract

Why do we listen to sad music? We seek to answer this question using a psychological approach. It is possible to distinguish perceived emotions from those that are experienced. Therefore, we hypothesized that, although sad music is perceived as sad, listeners actually feel (experience) pleasant emotions concurrent with sadness. This hypothesis was supported, which led us to question whether sadness in the context of art is truly an unpleasant emotion. While experiencing sadness may be unpleasant, it may also be somewhat pleasant when experienced in the context of art, for example, when listening to sad music. We consider musically evoked emotion vicarious, as we are not threatened when we experience it, in the way that we can be during the course of experiencing emotion in daily life. When we listen to sad music, we experience vicarious sadness. In this review, we propose two sides to sadness by suggesting vicarious emotion.

## Introduction

Some people listen to music to shift their mood, while others do so to alleviate feelings of depression, unhappiness, or emotional discomfort. We often regulate our mood by listening to our music of choice. People tend to listen to cheerful music when they want to improve their mood because specific qualities contained in that music amuse us and improve our emotional state. With this in mind, why do we listen to **sad music**? It is reasonable to assume that sad music would evoke sadness in listeners, as we tend to tap into the sadness it emits when we elect to listen to it.

KEY CONCEPT 1. Sad music Although music consists of many elements, including tempo, key, and melody, numerous previous studies have confirmed that existing musical pieces in minor keys sound sad to listeners (Hevner, 1935; Nielzén and Cesarec, 1982; Krumhansl, 1997; Peretz et al., 1998). Therefore, we focused on music in a minor key and defined it as sad music in this study.

However, this seems unusual. Within emotion psychology, sadness is generally regarded as an unpleasant emotion. According to the typical dimensional model of emotion suggested by Russell (1980), sadness is located in the position of displeasure and deactivating emotions (Russell, 2003). In addition, from an approach-avoidance perspective, people should want to avoid sadness. No one would be eager to experience sadness, being such an unpleasant emotion. This begs the question: why do we choose to listen to sad music when it should offer us an unpleasant experience?

Aristotle addressed this question by suggesting the concept of catharsis. If, as he suggested, sad music assuages depression, then it is not surprising that people would prefer sad music. Our recent study (Kawakami et al., 2013b) added an alternative idea explaining human preference for sad music. We suggested that people's ability to feel pleasure when listening to music that is perceived as sad might be related to a difference between the perception of emotion in the music and the emotion it actually evokes.

There are two types of emotions: perceived and felt. Perceived emotions concern what people perceive objectively, whereas felt emotions concern what people actually experience. It is possible to determine the affective aspects of a target's expression. We can usually recognize others' emotions using expressed cues including facial expression, tone of voice, and gestures. A similar process occurs when we listen to music, in that we recognize it as happy or sad using cues such as key, tempo, or volume. Conversely, we experience various emotions other than our objectively **perceived emotion**. When we look at an angry person, we can usually perceive that the person is angry, but we do not always experience anger ourselves; rather, we often experience fear in response to others' anger. Of course, when our experienced emotion is identical to our perceived emotion, then **felt emotion** and perceived emotion coincide.

KEY CONCEPT 2. Perceived emotion There are two kinds of emotions: perceived and felt (Gabrielsson, 2002). Perceived emotion refers to emotion that we perceive or recognize from our surroundings and environments. For example, when we listen to a piece of music that is being played, we are able to perceive it as being happy or sad.

KEY CONCEPT 3. Felt emotion Felt emotion refers to an emotion we actually experience. Indeed, perceived and felt emotions are identical in many cases. However, our earlier study (2013a) suggests that musically trained people experience pleasant emotions while listening to dissonance and music in minor keys despite perceiving them as unpleasant. Thus, there is a need to distinguish felt from perceived emotion.

Gabrielsson (2002) noted that it is essential to distinguish between perceived emotion and felt emotion, suggesting four patterns of relationships between these two kinds of emotions in response to music: positive relationship, negative relationship, no systematic relationship, and no relationship. A recent article (Schubert, 2013) reviewed these four relationships, and suggested that perceived and felt emotions are often coincident in many cases. For example, sad music is generally thought to make listeners feel sad, which would indicate a positive relationship between perceived and felt emotions (Table 1). Can this be expanded? Our earlier study (Kawakami et al., 2013a) presented a range of musical structures in which perceived emotion differed from felt emotion. The structures were composed of 21 musical stimuli, including key (major/minor), ascending (or descending) melody, and consonance (or dissonance). We found that, although dissonance and melody in a minor key were perceived as unpleasant sounds, listeners actually felt fewer unpleasant and more pleasant emotions when listening to these musical stimuli. Therefore, perceived emotion is not always congruent with felt emotion when related to particular musical structures. When felt emotion is opposed to perceived emotion, this is considered as a “negative relationship” (Table 1). Generally, as minor-key music is perceived as sad (Hevner, 1935), the above mentioned case, in which the minor-key melody evoked more pleasant emotions, exemplifies the common phenomenon of enjoyment of sad music. In our study, we attempted to clarify the underlying mechanisms of “pleasurable sadness” using this framework for negative relationships between perceived and felt emotions.

**Table 1:** **Relationships between perceived and felt emotions**.

	**Emotions**	
	**Perceived emotion**	**Felt emotion**
Positive relationship	Sad	Sad
Negative relationship	Sad	Pleasant

Further, Kawakami et al. (2013a) recently found that musically trained or experienced individuals perceived dissonant, minor-key music as unpleasant, but the stimuli did not evoke equally unpleasant emotions. As musically trained people have had more opportunity to listen to or play dissonant music than people who are not musically experienced, the emotion evoked by dissonant music was presumed to be influenced by musical experience. In light of this, in our study (Kawakami et al., 2013b), we hypothesized that musicians would experience more pleasant emotions than non-musicians would when listening to sad music, although both groups were expected to perceive an equal degree of sadness in the sound of the music.

## Method

### Participants

Twenty-five women and 19 men participated in the study. Participants were placed into one of two groups according to their musical experience. Professional musicians and college students who were majoring in music formed the “musician group” (*n* = 17), while individuals who were employed in fields unrelated to music or attended college and majored subjects other than music formed the “non-musician group” (*n* = 27). The participants' mean age was 25.3 years (*SD* = 6.6).

### Materials

We used the following three musical pieces: (1) Glinka's La Separation (F major and F minor), played at quarter note = 80; (2) Blumenfeld's Etude “Sur Mer” (G major and G minor), played at half note = 72; and (3) Granados's Allegro de Concierto (G major and G minor), played at quarter note = 70. Preparing both major- and minor-key versions for all musical pieces enabled us to examine whether perceived and felt emotions differed according to key. Kawakami et al. (2013b) present the scores.

We endeavored not to use prominent musical pieces as musical stimuli to avoid evoking particular memories that participants may have associated with well-known music, thereby ensuring that emotion evoked by the music would be derived from the stimuli rather than a memory. We asked participants if they recognized the music, and no one reported having heard the musical stimuli before.

### Self-report measures

Participants reported how they perceived the music (their perception of the music) and how it made them feel (their own emotional state) using 62 emotion-related descriptive words and phrases on a scale ranging from 0 (not at all) to 4 (very much). These descriptive words and phrases referred to various types of emotion that had been used in earlier studies (Hevner, 1936; Taniguchi, 1995; Zentner et al., 2008). For more details, refer to Table 1 in Kawakami et al. (2013b).

### Procedure

The participants performed four tasks. The first asked participants to listen to the music (major or minor) and report either their perceived or felt emotion. The second required that participants listen to the music played in a different key to that used in the first piece and report their perceived or felt emotion according to which of these they reported in the first task (i.e., the same type of emotion, either perceived or felt, was reported in both tasks). In the third and fourth tasks, participants repeated tasks 1 and 2 but gave an indication of the alternate type of emotion (perceived or felt).

We used a traditional question to evaluate listeners' feelings in response to the music (felt emotion): “How did you feel when listening to this musical stimulus?” We evaluated each listener's perceived emotion with the following question: “How would normal people feel when listening to this musical stimulus?” Though this method may appear to be an inadequate measure of perceived emotion, we presumed the following process: “This music sounds sad to me and most likely to other people as well. I cannot know how other people actually feel, but my best guess is that they will feel the emotion that the music portrays.”

### Statistical analysis

To determine the characteristics of relevant factors, we performed an exploratory factor analysis to classify the 62 emotion-related descriptive words and phrases included using 176 datasets: 2 (perceived/felt emotion) × 2 (major/minor key) × 44 participants. For each resultant factor, we conducted a Three-Way analysis of variance (ANOVA) with the following design: musical emotion (perceived vs. felt) × key (major vs. minor) × musical experience (musicians vs. non-musicians). This allowed us to compare these variables for each factor.

## Results

The 62 emotion-related descriptive words and phrases were investigated via factor analysis, and four factors were extracted: “tragic emotion,” “heightened emotion,” “romantic emotion,” and “blithe emotion.” We then conducted a Three-Way ANOVA for each factor.

### Perceived and felt emotions in response to sad music

**Figure 1:**
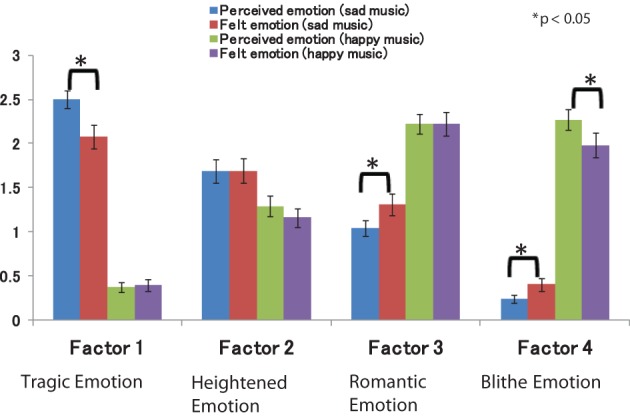
**Mean factor scores for perceived and felt emotions**.

We tested our hypothesis that felt emotion would not necessarily correspond with perceived emotion, particularly in response to music played in a minor key. Our results showed that, although the sad music was perceived as more tragic, participants did not experience the corresponding tragic emotions (e.g., gloomy, meditative, and miserable). Mean factor scores were 2.50 for perceived tragic emotion and 2.08 for felt tragic emotion. Indeed, participants felt more romantic (e.g., fascinated, dear, and in love) and blithe emotions (e.g., merry, animated, and feel like dancing) than they perceived such emotions when listening to the same sad music. Figure 1 represents mean factor scores for perceived and felt emotion ratings; mean factor scores were 1.04 for perceived romantic emotion, 1.31 for felt romantic emotion, 0.24 for perceived blithe emotion, and 0.40 for felt blithe emotion.

### The effects of musical training

Participants' emotional responses were not associated with musical training. Music that was perceived as tragic evoked fewer sad and more romantic emotions in both musicians and non-musicians. Therefore, our hypothesis—when participants listened to sad (i.e., minor-key) music, those with more musical experience (relative to those with less experience) would feel more pleasant emotions than they would perceive—was not supported. According to an earlier study (Kawakami et al., 2013a), musically experienced participants judged perceived emotion as more unpleasant than felt emotion when exposed to very short minor-key musical stimuli (played for approximately 5 s). However, they also felt fewer unpleasant emotions and more pleasant feelings when listening to music in a minor key. The lack of significant differences between musicians and non-musicians in terms of emotional reactions to sad music in the current study could be a function of the musical stimuli that we used. Although Kawakami et al. (2013a) used a musical structure that lacked ecological validity (each stimulus was presented for approximately 5 s), we used existing musical pieces (presented for approximately 30 s) that included a number of musical structures (Table 2), which allowed participants to capture more esthetic information from the music. Consequently, regardless of their musical experience, participants may have reacted to the esthetic aspects of the sad music, which would render musical experience ineffectual. This explanation seems plausible considering that people, including those with musical experience, habitually enjoy sad music.

**Table 2:** **Effect of musical training on the difference between perceived and felt emotions**.

	**Kawakami etal. (2013a)**	**Kawakami etal. (2013b)**
Musical stimuli	Single musical structure (5.06 s for each)	Excerpt of existing music (33.8 s for each)
Difference between perceived and felt emotions	Only suitable for people with high levels of musical training	Suitable for both musicians and non-musicians

Listening to sad music evokes ambivalent emotions: both positive and negative relationships.

With regard to felt emotion, sad music evoked both tragic and romantic emotions in participants. Romantic emotion included emotions such as feeling fascinated, dear, and in love, and it would be appropriate to regard these emotions as pleasant rather than unpleasant. Therefore, when listening to sad music, it would appear that people experience ambivalent emotion. Aristotle's explanation for a preference for sad music was based on the belief that sad music evoked sad emotion. In contrast, the results of our study suggest that sad music evokes other emotions in addition to tragic (i.e., sad) emotion and preference for sad music is based on positive emotions. It is therefore possible to love sad music because of the positive emotional components it evokes, which may relieve supporters of sad music.

Having established that we experience ambivalent emotions when listening to sad music, why does this occur? The positive relationship suggested by Gabrielsson (2002) appears to be the simplest explanation for the relationship between perceived and felt emotions and how sad music evokes negative emotion in listeners; however, the majority of previous studies have not distinguished between perceived and felt emotion. More precisely, results showing that people experience ambivalent emotions in response to sad music demonstrate that perceived and felt emotions in response to sad music are not coincident. The phenomenon of enjoying sad music indicates that both negative and positive relationships exist between perceived and felt emotions. We discuss this phenomenon using three approaches in the following section.

## Discussion

We offer three approaches to explain why both pleasant and unpleasant emotions are evoked by sad music. The sweet anticipation and re-evaluation approaches presume that sadness evoked by sad music is unpleasant and identical to common emotions, as posited by Juslin (2013). Using these approaches, it would be possible to explain why sad music evoked stronger felt romantic emotion than perceived romantic emotion. We also introduce the notion of “**vicarious emotion**.” This approach suggests that sadness evoked by sad music is qualitatively different from sadness experienced in daily life. It would be of help to explain why sad music evoked weaker felt tragic emotion than perceived tragic emotion. However, it should be noted that these three theories are not mutually exclusive.

KEY CONCEPT 4. Vicarious emotion We defined vicarious emotion as that experienced while listening to music. Although emotions we experience in daily life are often derived from actual objects or situations, vicarious emotions are free from the essential unpleasantness of their genuine counterparts. As listening to music poses no direct threat or danger to us, we can experience a range of vicarious emotions in such a situation.

### Sweet anticipation

While listening to music, we tend to anticipate what is coming next. According to Meyer's (1956) musical expectancy theory, a violation, delay, or confirmation of a listener's expectations evokes emotion, which is derived from a prediction of the continuation of the music (Huron, 2006). A listener's expectation would be confirmed if the predicted sound was heard, which would evoke positive emotion (i.e., “sweet anticipation”).

Therefore, even if listeners experience sadness when listening to sad music, this sweet anticipation allows them to feel positive emotions simultaneously. Consequently, the pleasant experience would have arisen as a consequence of sweet anticipation.

### Re-evaluation in the context of art

The role of the emotion-appraisal process is considered crucial in the two-factor theory of emotion (Schachter and Singer, 1962) and cognitive-mediational theory (Lazarus, 1966, 1993). According to Oatley and Johnson-Laird (2014), this is the case with three representative cognitive theories of emotion: the action-readiness theory of emotions (Frijda, 1986, 2007; Frijda and Parrott, 2011); the core-affect theory of emotions (Russell, 2003); and the communicative theory of emotions (Oatley and Johnson-Laird, 1987, 2011). It is also essential for people to understand their own situations to experience emotion (Lazarus, 1966). When we listen to music, being in a listening situation is obvious to us; therefore, how emotion is evoked would be influenced by our cognitive appraisal of listening to music. For example, a cognitive appraisal of listening to sad music as engagement with art would promote positive emotion, regardless of whether that music evoked feelings of unpleasant sadness, thereby provoking the experience of ambivalent emotions in response to sad music. It is possible that we initially experience negative emotion, such as sadness, and subsequently experience positive emotion because of the rewarding effects of enjoying art (Koelsch, 2012).

This approach may resemble those of Schubert (1996) and Juslin (2013), in that both consider esthetic context or judgment as causes of pleasant emotion. Schubert (1996) suggests that esthetic context activates a node in the neural network that deactivates the displeasure center of the brain. This is based on the Berlyne's (1971) notion that there are cues that inhibit the aversion system in an artistic context. Juslin (2013) suggested that conflicting emotion, such as sadness and pleasure, originates from the “emotional contagion” and “esthetic judgment” mechanisms. He explains that sadness elicited by music arises through emotional contagion, and that the pleasure elicited by music occurs in response to our perception of the beauty of the music. As these two mechanisms occur concurrently, it is possible to experience pleasurable sadness when we listen to sad music.

The ideas expressed by Schubert and Juslin are interesting, and our results indicate that participants experienced both tragic and romantic emotion when listening to sad music. However, notably, participants experienced less tragic emotion than they perceived when listening to sad music. If tragic emotion was evoked through the emotional contagion mechanism, the degree of tragic emotion experienced could be expected to be equal to or surpass the listener's perceived emotion in response to sad music. Because the emotional contagion emanates from others, the perceived emotion in this study measured the emotional states of others.

In addition, so-called esthetic emotion would be comparable to the romantic emotion in our experiment. Interestingly, participants experienced more romantic emotion than they perceived when listening to sad music. When participants listened to happy music, this tendency was absent, and the degree of romantic emotion perceived did not differ significantly from the degree of romantic emotion actually experienced. Therefore, pleasant emotion may originate not only from esthetic judgment but also from a lack of real danger. As listening to music was not related to an actual threat to their safety, participants would experience less tragic emotion and more romantic emotion than they perceived.

### Vicarious emotions in relation to art

Is the sadness evoked by music really an unpleasant emotion? Is it similar to the sad emotion experienced in daily life? In many cases, emotions evoked by music are not regarded as different from those experienced in daily life and listeners frequently report basic emotions (e.g., happy, sad, or angry) in response to music. In our study, participants also reported sadness, but we suspect that the quality of that sadness differs from sadness in daily life in that we are willing to experience it, whereas we are not generally willing to experience sadness in daily life; this indicates that sadness evoked by music is pleasant and the same emotion experienced in daily life is not. Indeed, participants might only have chosen “sadness” as a description of their emotion because the word was selected by the experimenter for the questionnaire. Hence, it may be too soon to conclude that sadness is only related to unpleasant emotion.

Of course, we agree that music evokes similar emotions to those experienced in everyday life in cases in which personal memories, such as those of a broken relationship or bereavement, are associated with the music. In such cases, listeners would experience unpleasant sadness similar in quality to that experienced in daily life. In this situation, the sadness evoked by the music is, to be exact, derived from the memory associated with the music rather than from the music itself, which would merely serve as a trigger for the unpleasant sadness related to the memory.

**Figure 2:**
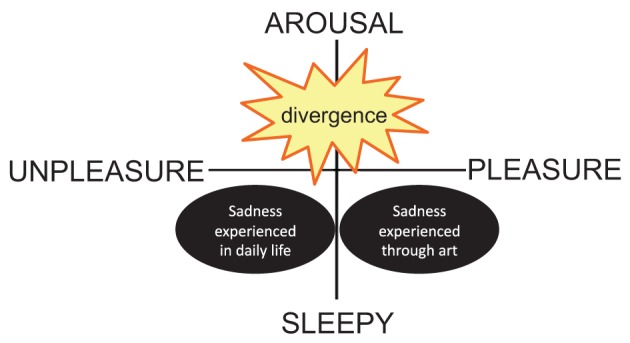
**The two kinds of sadness**.

In contrast, some researchers have denied that music can evoke common “everyday emotions” (e.g., sadness, happiness, or anger; Kivy, 1990; Konečni, 2003; Scherer, 2003). Noy (1993) noted that “the emotion evoked by music are not identical with the emotion aroused by everyday interpersonal activity” (p. 126). As Eerola and Vuoskoski (2011) noted, although sadness is generally considered an unpleasant emotion, in the context of music, it may not be classified as unpleasant. That is, in the context of art, emotion-evoking processes may differ from those of day-to-day emotions. Therefore, as Scherer (2004) proposed when discussing the distinction between goal-oriented utilitarian emotion and esthetic emotion, the sadness that we experience while listening to sad music may differ from that experienced in daily life. As shown in Figure 2, we imagine that, although sadness experienced in daily life would be mapped in the displeasure area, sadness experienced via art would be mapped in the pleasure area. That is, the two types of sadness may diverge, although sadness is typically considered as a solely unpleasant emotion.

In sum, we consider emotion experienced in response to music to be qualitatively different from emotion experienced in daily life; some earlier studies (Scherer and Zentner, 2001, 2008; Scherer, 2003; Zentner et al., 2008) also proposed that music may evoke music-specific emotions. The difference between the emotions evoked in daily life and music-induced emotions is the degree of directness attached to emotion-evoking stimuli. Emotion experienced in daily life is direct in nature because the stimuli that evoke the emotion could be threatening. However, music is a safe stimulus with no relationship to actual threat; therefore, emotion experienced through music is not direct in nature. The latter emotion is experienced via an essentially safe activity such as listening to music. We call this type of emotion “vicarious emotion.”

As emotions evoked by music have no real threat attached, we suppose that a wide variety and intensity of emotions could be considered vicarious emotions. However, it might be difficult to apply the vicarious emotion hypothesis to particular emotions or deep sadness associated with non-musical influences; for example, where extremely intense sadness is related to a non-musical factor, such as the death of a close relative. With regard to listening to music, various types of sadness could be considered vicarious emotions because we assume that musically evoked sadness does not actually pose a threat, regardless of the intensity of that sadness.

**Figure 3:**
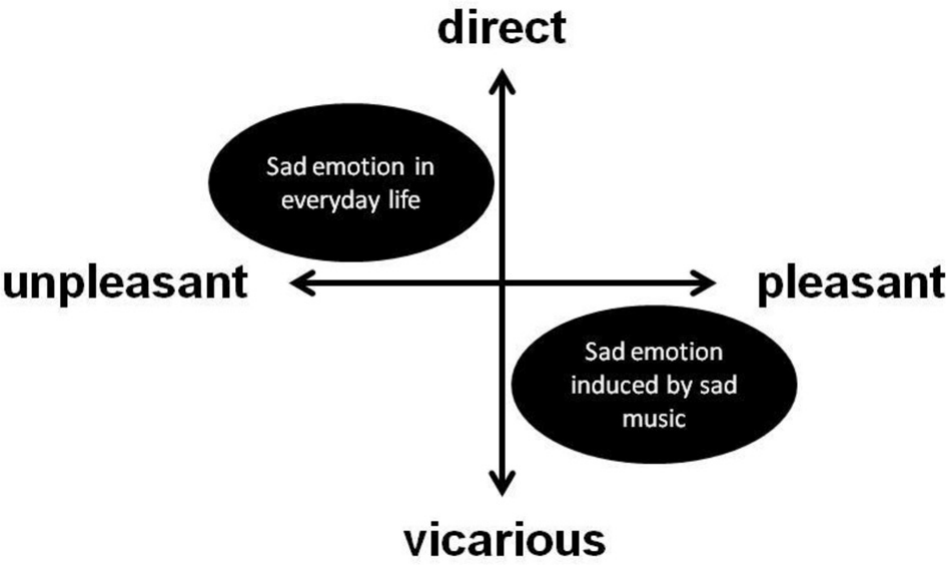
**Pleasant-unpleasant, direct-vicarious model**.

We added a direct-vicarious axis to the pleasant-unpleasant axis, as shown in Figure 3. The horizontal axis (pleasant-unpleasant) depicts the emotional evaluation of experienced emotion, and the vertical axis (direct-vicarious) depicts the way in which individuals relate to the stimuli that elicit the emotion. Because of their direct relationship to the cause, emotions experienced in daily life would be located in the first and second quadrants in Figure 3. Conversely, emotions experienced while listening to music would be located in the fourth quadrant because despite inducing emotion, music has no direct relationship to the listener; therefore, listeners experience vicarious emotions in response to music, which enables them to experience pleasant emotion even when they perceive the music as sad.

Focusing on vicarious emotion could improve our understanding of the nature of the experience of art and highlight an aspect of our emotional system that is sensitive to more than just food or threats. When we pine or weep at the beauty of sad music, we experience an astounding range of emotions. An endeavor to understand what such emotional functions provide could become a quest for the meaning and significance of art (Kawakami, 2013).

## Conclusion

Measuring both perceived and felt emotions, we clarified the phenomenon of enjoyment in listening to sad music. We hypothesized that sad music would be perceived as sad, but the experience of listening to sad music would evoke positive emotions in listeners. The results supported our hypothesis, in that although sad music was perceived as more tragic, listeners experienced less tragic emotion. Listeners also experienced romantic emotion; therefore, we could argue that sad music inspired ambivalent emotions in the listeners. Moreover, we suggest that the reason people experience ambivalent emotions when listening to sad music may be that the music generates vicarious emotions in listeners. That is, even if the music evokes a negative emotion, listeners are not faced with any real threat; therefore, the sadness that listeners feel has a pleasant, rather than an unpleasant, quality to it. This suggests that sadness is multifaceted, whereas it has previously been regarded as a solely unpleasant emotion.

## Future issues

Although we used minor-key musical pieces as sad music in our study (Kawakami et al., 2013b), examination of other musical elements, including acoustic parameters (Gingras et al., 2013), is required. Moreover, we are anxious to know more why minor-key music sounds sad and evokes sadness in listeners; we believe this to be an important issue.

### Conflict of interest statement

The authors declare that the research was conducted in the absence of any commercial or financial relationships that could be construed as a potential conflict of interest.
